# An Increase in Mean Platelet Volume from Baseline Is Associated with Mortality in Patients with Severe Sepsis or Septic Shock

**DOI:** 10.1371/journal.pone.0119437

**Published:** 2015-03-05

**Authors:** Chan Ho Kim, Seung Jun Kim, Mi Jung Lee, Young Eun Kwon, Yung Ly Kim, Kyoung Sook Park, Han Jak Ryu, Jung Tak Park, Seung Hyeok Han, Tae-Hyun Yoo, Shin-Wook Kang, Hyung Jung Oh

**Affiliations:** 1 Department of Internal Medicine, International St. Mary’s Hospital, Catholic Kwandong University College of Medicine, Incheon, Korea; 2 Department of Medicine, Graduate School of Medicine, Yonsei University, Seoul, Korea; 3 Department of Internal Medicine, College of Medicine, Yonsei University, Seoul, Korea; 4 Brain Korea 21 PLUS Project for Medical Science, Yonsei University, Seoul, Korea; Azienda Ospedaliero-Universitaria Careggi, ITALY

## Abstract

**Introduction:**

Mean platelet volume (MPV) is suggested as an index of inflammation, disease activity, and anti-inflammatory treatment efficacy in chronic inflammatory disorders; however, the effect of MPV on sepsis mortality remains unclear. Therefore, we investigated whether the change in MPV between hospital admission and 72 hours (ΔMPV_72h-adm_) predicts 28-day mortality in severe sepsis and/or septic shock.

**Methods:**

We prospectively enrolled 345 patients admitted to the emergency department (ED) who received standardized resuscitation (early goal-directed therapy) for severe sepsis and/or septic shock between November 2007 and December 2011. Changes in platelet indices, including ΔMPV_72h-adm_, were compared between survivors and non-survivors by linear mixed model analysis. The prognostic value of ΔMPV_72h-adm_ for 28-day mortality was ascertained by Cox proportional hazards model analysis.

**Results:**

Thirty-five (10.1%) patients died within 28 days after ED admission. MPV increased significantly during the first 72 hours in non-survivors (*P* = 0.001) and survivors (*P* < 0.001); however, the rate of MPV increase was significantly higher in non-survivors (*P* = 0.003). Nonetheless, the difference in the platelet decline rate over the first 72 hours did not differ significantly between groups (*P* = 0.360). In multivariate analysis, ΔMPV_72h-adm_ was an independent predictor of 28-day mortality, after adjusting for plausible confounders (hazard ratio, 1.44; 95% confidence interval, 1.01–2.06; *P* = 0.044).

**Conclusions:**

An increase in MPV during the first 72 hours of hospitalization is an independent risk factor for adverse clinical outcomes. Therefore, continuous monitoring of MPV may be useful to stratify mortality risk in patients with severe sepsis and/or septic shock.

## Introduction

The mean platelet volume (MPV) describes the average size of platelets in a blood sample and is routinely measured by automated hematology analyzers using either electrical impedance or optical fluorescence method [1,2]. Increased platelet volume and size reflects the existence of a thrombotic and inflammatory milieu; thus, MPV is suggested as a possible marker of platelet function and activation [3–5]. Over the past decade, several studies have shown that increased MPV is an independent risk factor for cardio- and cerebrovascular diseases and is associated with poor clinical outcomes of these diseases [6–10]. Additionally, MPV has been considered an index for inflammation, disease activity, and efficacy of anti-inflammatory treatment in several chronic inflammatory disorders, such as inflammatory bowel disease, rheumatoid arthritis, and ankylosing spondyloarthritis [11–15].

The time course of platelet counts and its function in critically ill patients, especially in patients with sepsis, have been elucidated by several previous studies [16–19]. Although the underlying mechanism is not yet completely understood, the sophisticated interaction of platelets with pathogens and endothelial cells may culminate in sepsis, a severe pathophysiologic cascade characterized by significant reductions in platelet counts and platelet dysfunction [17,18,20,21]. Only a few studies have revealed the relationship between MPV and prognosis in infectious diseases, including sepsis [22–24]. Moreover, little is known about the potential influence of MPV and its change on mortality in a homogenous group of patients with sepsis. Therefore, we investigated whether the change in MPV between baseline and 72 hours after hospital admission has prognostic value for clinical outcomes in severe sepsis and/or septic shock.

## Materials and Methods

### Patients

Eligible adult patients admitted to the emergency department (ED) with the clinical features of severe sepsis and/or septic shock between November 2007 and December 2011 were assessed for possible enrollment according to inclusion and exclusion criteria. Since November 2007, early goal-directed therapy (EGDT) has been implemented in the intensive care unit and ED of our institution as part of a quality improvement initiative. If a patient presented with two or more systemic inflammatory response syndrome criteria and had a suspicious sign of infection, his or her eligibility for EGDT was assessed. One or both of the following trigger the initiation of the EGDT protocol: (a) initial systolic blood pressure < 90 mmHg despite a 20 mL/kg intravenous crystalloid fluid challenge or (b) initial serum lactate level ≥ 4 mmol/L. Study exclusion criteria were (a) age < 18 years, (b) any contraindication to central venous catheterization, and/or (c) presence of a do-not-resuscitate order [25].

**Fig 1:**
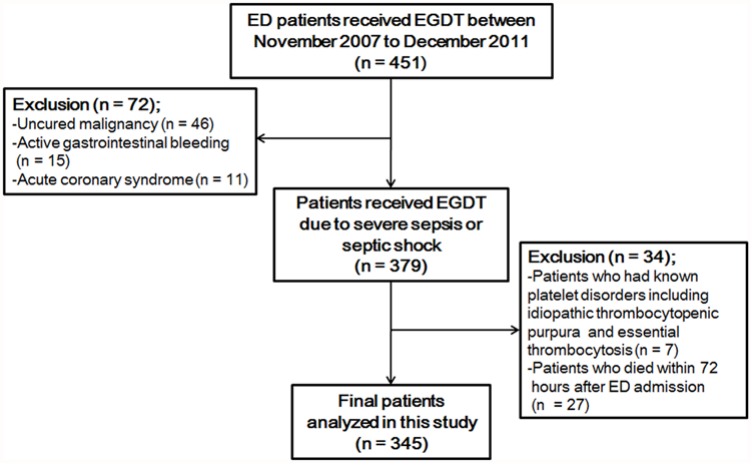
Flow diagram of study patients. From November 2001 to December 2011, 451 patients who received early-goal directed therapy (EGDT) in the emergency department (ED) were assessed for possible enrollment according to inclusion and exclusion criteria, and 345 patients were included in the final analysis.

A total of 451 patients who received EGDT in the ED were initially enrolled in the study. Seventy-two were excluded because of uncured malignancy, active gastrointestinal bleeding, and acute coronary syndrome. Seven patients with known platelet disorders, such as idiopathic thrombocytopenic purpura and essential thrombocytosis, were also excluded. The final analysis included 345 patients, excepting 27 patients who died within 72 hours after ED admission (Fig. 1).

The study protocol was approved by the Institutional Review Board of the Yonsei University Health System Clinical Trial Center. All patients provided written informed consent to participate in the study.

### Data collection

Baseline characteristics, including demographic information and comorbidities, were collected. The Charlson Comorbidity Index was used to assess the burden of chronic disease [26,27]. For disease severity assessment, both the Acute Physiology and Chronic Health Evaluation II (APACHE II) score and the Sequential Organ Failure Assessment (SOFA) score were determined according to the worst values within the initial 24 hours of ED admission. The SOFA score was calculated from the following parameters: arterial partial pressure of oxygen/fraction of inspired oxygen, platelet count, bilirubin, blood pressure and use of an inotropic agent, Glasgow Coma Scale score, and creatinine level or urine output. In addition, MPV, platelet count, white blood cell (WBC) count, and hemoglobin (Hb) level were measured at initial presentation and at 36 and 72 hours after ED admission. Venous blood samples for laboratory counts were collected from all patients in tubes containing ethylenediamine tetra-acetic acid (EDTA) and analyzed with an Advia 2120 hematology analyzer (Siemens Healthcare Diagnostics, Deerfield, IL) within 30 minutes of sample collection. The normal reference range for MPV in our hospital laboratory is 7.4 to 10.4 fL.

### Definitions

Sepsis, severe sepsis, and septic shock were defined based on American College of Chest Physicians/Society of Critical Care Medicine consensus conference definitions [28]. Sepsis was defined by two or more of the following conditions resulting from infection: (i) temperature greater than 38°C or less than 36°C, (ii) heart rate greater than 90 beats/min, (iii) respiratory rate greater than 20 breaths/min or arterial carbon dioxide tension less than 32 mmHg, and (iv) WBC count greater than 12,000 cells/mm^3^ or less than 4,000 cells/mm^3^. Severe sepsis was defined as sepsis associated with organ dysfunction, hypoperfusion abnormality, or sepsis-induced hypotension. Hypoperfusion abnormalities included lactic acidosis, oliguria, and acute alteration of mental status. In addition, septic shock was defined as sepsis with hypotension, despite adequate fluid resuscitation. Hypotension was defined as a systolic blood pressure of 90 mmHg or less, or a reduction of greater than 40 mmHg from baseline in the absence of other causes of low blood pressure.

Infection site was categorized as pneumonia, peritonitis, urinary tract infection, exacerbation of chronic obstructive pulmonary disease, catheter-related infection, primary bacteremia (excluding untreated Staphylococcus epidermidis bacteremia), miscellaneous sites (mediastinitis, prostatitis, osteomyelitis, etc.), or multiple sites [29]. Moreover, effectiveness of antibiotics was assessed on the basis of microbial culture results, the known susceptibility of the organism to the antimicrobial agents used, and antimicrobial susceptibility test [29].

### Statistical analyses

Continuous variables are expressed as mean ± standard deviation, and categorical variables as numbers with percentages. We evaluated 28-day all-cause mortality as a primary endpoint. Patients who died within 28 days after ED admission were defined as ‘non-survivors.’ Baseline characteristics are presented according to the occurrence of the primary outcome (survivors versus [vs.] non-survivors) and were compared between the 2 groups using Student’s *t*-test for continuous variables and chi-square test for categorical variables. Changes in platelet indices including MPV and platelet count during the first 72 hours after ED admission were compared between ‘survivors’ and ‘non-survivors’ by linear mixed model analysis. In our implement of the mixed model method, the intercept and the regression coefficient for follow-up time were treated as random effects such that each patient had a unique intercept and regression coefficient. The *post-hoc* analysis was performed with Bonferroni correction. MPV at 72 hours minus MPV at baseline was considered the change in MPV at 72 hours after ED admission (ΔMPV_72h-adm_). The relationship between ΔMPV_72h-adm_ and other demographic/biochemical parameters was assessed with Pearson’s correlation analysis. The prognostic value of ΔMPV_72h-adm_ for 28-day mortality was ascertained by Cox proportional hazards model, and the results are presented as hazard ratios (HRs) with 95% confidence intervals (CIs). Covariate selection for the multivariate Cox model was based on *P*-value < 0.1 in univariate analysis. All tests were two-sided, and a *P*-value of < 0.05 was considered statistically significant. Statistical analyses were performed with SPSS for Windows version 19.0 (IBM Corporation, Chicago, IL).

## Results

### Baseline characteristics

The mean age of the patients was 64.2±15.7 years, and 169 (49.0%) were male. The mean APACHE II score was 17.4±7.2, and the mean SOFA score was 8.0±2.8. In addition, MPV ranged from 6.7 to 15.0 fL (median, 8.4 fL; mean, 8.64 fL) at baseline and from 6.6 to 14.7 fL (median, 8.7 fL; mean, 8.96 fL) at 72 hours after ED admission. The main infection sites were urinary tract (25.2%) and lung (24.1%) followed by intra-abdominal cavity (22.0%) (Table 1). The baseline demographic, clinical, and biochemical data of each group stratified by 28-day all-cause mortality are presented in Table 1. As expected, non-survivors exhibited significantly higher APACHE II and SOFA scores and C-reactive protein (CRP) and lactate levels than did survivors, whereas body mass index (BMI); platelet count; estimated glomerular filtration rate (eGFR); and albumin, total cholesterol, and pH levels in non-survivors were significantly lower than those in survivors. The infection sites of survivors were relatively evenly distributed compared with those of non-survivors, whose infection site was mainly the lungs (45.7%). Moreover, the proportions of patients who received renal replacement therapy (RRT) and platelet transfusion were significantly higher in non-survivors. However, there were no significant differences in age, mean arterial pressure, Charlson Comorbidity Index, WBC, Hb, serum creatinine, total bilirubin, bicarbonate, RBC transfusion, and heparin use between the two groups (Table 1).

**Table 1:** Baseline clinical characteristics and biochemical variables according to the occurrence of 28-day all-cause mortality.

Variable	Total (*n* = 345)	Survivors (*n* = 310)	Non-survivors∮ (*n* = 35)	*P*-value§
**Demographic data**				
Age (years)	64.2 ± 15.7	63.7 ± 15.9	68.9 ± 13.0	0.060
Male sex, n (%)	169 (49.0%)	144 (46.5%)	25 (71.4%)	0.005
MAP (mmHg)	59.9 ± 8.7	59.9 ± 8.9	60.5 ± 7.2	0.693
Body mass index (kg/m^2^)	23.0 ± 3.9	23.1 ± 4.0	21.8 ± 2.6	0.016
APACHE II score	17.4 ± 7.2	16.5 ± 6.6	25.9 ± 6.8	<0.001
SOFA score	8.0 ± 2.8	7.7 ± 2.6	11.1 ± 3.0	<0.001
Charlson Comorbidity Index	1.4 ± 1.4	1.4 ± 1.5	1.4 ± 1.4	0.804
**Biochemical data**				
WBC (× 10^3^/mm^3^)	14.1 ± 9.3	13.8 ± 8.5	17.1 ± 14.8	0.211
Hemoglobin (g/dL)	12.3 ± 2.2	12.3 ± 2.2	12.0 ± 2.1	0.471
Platelet (× 10^3^/mm^3^)	209.1 ± 118.9	214.6 ± 118.1	160.7 ± 116.8	0.011
MPV at baseline (fL)	8.64 ± 1.20	8.54 ± 1.10	9.54 ± 1.66	0.001
MPV at 72 hours (fL)	8.96 ± 1.19	8.80 ± 1.01	10.35 ± 1.69	<0.001
ΔMPV_72h-adm_ (fL)†	0.32 ± 0.95	0.26 ± 0.89	0.80 ± 1.30	0.021
CRP (mg/dL)	15.3 ± 11.4	14.7 ± 11.2	20.6 ± 11.5	0.005
Creatinine (mg/dL)	2.1 ± 1.8	2.0 ± 1.8	2.4 ± 1.5	0.221
eGFR (mL/min/1.73 m^2^)	53.3 ± 27.7	54.6 ± 28.0	41.4 ± 21.4	0.002
Albumin (g/dL)	3.3 ± 0.7	3.4 ± 0.7	2.6 ± 0.7	<0.001
Total cholesterol (mg/dL)	127.9 ± 42.8	130.9 ± 41.2	101.9 ± 48.0	<0.001
Total bilirubin (mg/dL)	1.2 ± 1.5	1.2 ± 1.3	1.9 ± 2.5	0.105
pH	7.42 ± 0.09	7.43 ± 0.09	7.37 ± 0.14	0.010
Bicarbonate (mEq/L)	21.1 ± 5.2	21.2 ± 5.1	20.6 ± 6.1	0.093
Lactate (mmol/L)	3.52 ± 2.91	3.31 ± 2.63	5.36 ± 4.38	0.010
**Infection site, n** (%)				<0.001
Lung (pneumonia)	83 (24.1%)	67 (21.6%)	16 (45.7%)	
Urinary tract	87 (25.2%)	85 (27.4%)	2 (5.7%)	
Intra-abdominal site	76 (22.0%)	76 (24.5%)	0 (-)	
Other	73 (21.2%)	61 (19.7%)	12 (34.3%)	
Multiple sites	26 (7.5%)	21 (6.8%)	5 (14.3%)	
**Platelet transfusion, n (%)‡**	26 (7.5%)	17 (5.5%)	9 (25.7%)	<0.001
**RBC transfusion, n (%)‡**	22 (6.4%)	19 (6.1%)	3 (8.6%)	0.478
**Heparin, n (%)‡**	8 (2.3%)	7 (2.3%)	1 (2.9%)	0.579
**Acute kidney injury, n (%)¶**	186 (53.9%)	161 (51.9%)	25 (71.4%)	0.028
**RRT, n (%)**	56 (16.2%)	38 (12.3%)	18 (51.4%)	<0.001

Data are mean ± standard deviation or *n* (%). MAP, mean arterial pressure; APACHE II, Acute Physiology and Chronic Health Evaluation II; SOFA, Sequential Organ Failure Assessment; WBC, white blood cell; MPV, mean platelet volume; CRP, C-reactive protein; eGFR, estimated glomerular filtration rate; RBC, red blood cell; RRT, renal replacement therapy.

∮Patients who died within 28 days after emergency department admission.

§*P* value comparisons between survivors and non-survivors.

†ΔMPV_72h-adm_ was calculated as MPV at 72 hours—MPV at baseline.

‡Patients who received platelet transfusion, RBC transfusion, or heparin within 72 hours after admission.

¶Acute kidney injury was defined as any of the following: (a) increase in serum creatinine level by ≥ 0.3 mg/dL within 48 h; (b) increase in serum creatinine level to ≥ 1.5 times baseline, which is known or presumed to have occurred within the prior 7 days; (c) urine volume < 0.5 mL/kg/h for 6 h.

### Trends in the platelet indices during the first 72 hours

**Fig 2:**
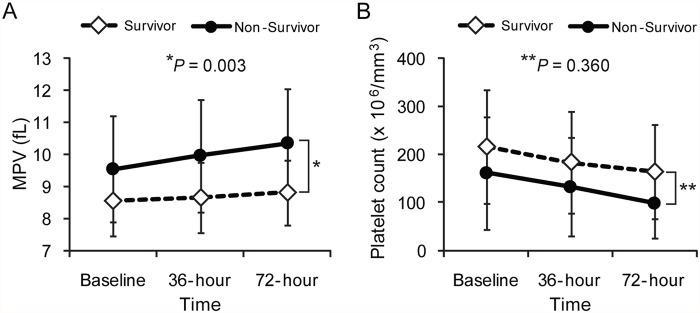
Comparison of trends in the platelet indices between survivors and non-survivors during the first 72 hours after emergency department admission. (A) The rate of mean platelet volume increase over the first 72 hours in non-survivors was significantly different from that observed in survivors (*P* = 0.003). (B) The rate of platelet count decline over the first 72 hours was comparable between the 2 groups (*P* = 0.360).

The trends in the platelet indices during the first 72 hours after ED admission are shown in Fig. 2 and Table 2. Non-survivors exhibited a significantly higher baseline MPV than survivors (9.54±1.66 vs. 8.54±1.10; *P* = 0.001). MPV levels increased significantly during the first 72 hours in both non-survivors (*P* = 0.001) and survivors (*P* < 0.001). However, the linear mixed model revealed a significantly increased rate of MPV over the first 72 hours in non-survivors than in survivors (*P* = 0.003) (Fig. 2A). In addition, ΔMPV_72h-adm_, defined as MPV at 72 hours—MPV at baseline, was significantly greater in non-survivors than in survivors (0.80±1.30 vs. 0.26±0.89 fL; *P* = 0.021). In contrast, although platelet counts decreased significantly during the first 72 hours in both groups (*P* < 0.001), a linear mixed model showed no significant difference in the rate of platelet count decline over the first 72 hours between groups (*P* = 0.360) (Fig. 2B).

**Table 2:** Trends in the platelet indices during the first 72 hours.

Variable	Baseline	At 36 hours	At 72 hours
**MPV (fL)**			
**Survivors**	8.54 ± 1.10	8.65 ± 1.10	8.80 ± 1.01
**Non-survivors**	9.54 ± 1.66	9.96 ± 1.76	10.35 ± 1.69
**Platelet counts (x 10** ^**3**^ **/mm** ^**3**^ **)**			
**Survivors**	214.6 ± 118.1	182.9 ± 105.9	162.9 ± 98.2
**Non-survivors**	160.7 ± 116.8	132.2 ± 101.6	97.8 ± 72.6

Data are mean ± standard deviation. MPV, mean platelet volume.

### Correlation between changes in MPV and other parameters

Pearson’s correlation analysis revealed a significant inverse correlation between ΔMPV_72h-adm_ and eGFR (r = -0.128; *P* = 0.021) (Table 3). In contrast, age, APACHE II score, and CRP showed significant positive associations with ΔMPV_72h-adm_ (r = 0.161; *P* = 0.003, r = 0.178; *P* = 0.001, r = 0.131; *P* = 0.016, respectively) (Table 3).

**Table 3:** Correlation between ΔMPV_72h-adm_ and variables.

Variable	ΔMPV_72h-adm_	
	*r*	*P*-value
**Age (years)**	0.161	0.003
**Body mass index (kg/m** ^**2**^ **)**	0.033	0.538
**APACHE II score**	0.178	0.001
**SOFA score**	0.018	0.746
**WBC (× 10** ^**3**^ **/mm** ^**3**^ **)**	-0.053	0.331
**Hemoglobin (g/dL)**	0.054	0.320
**Platelet (× 10** ^**3**^ **/mm** ^**3**^ **)**	-0.041	0.443
**CRP (mg/dL)**	0.131	0.016
**Creatinine (mg/dL)**	0.004	0.940
**eGFR (mL/min/1.73 m** ^**2**^ **)**	-0.128	0.021
**Albumin (g/dL)**	0.018	0.737
**Total cholesterol (mg/dL)**	0.030	0.582
**Total bilirubin (mg/dL)**	0.013	0.815
**Lactate (mmol/L)**	0.039	0.471

ΔMPV_72h-adm_, mean platelet volume at 72 hours—mean platelet volume at baseline; APACHE II, Acute Physiology and Chronic Health Evaluation II; SOFA, Sequential Organ Failure Assessment; WBC, white blood cell; CRP, C-reactive protein; eGFR, estimated glomerular filtration rate.

### Increase in MPV and all-cause mortality

**Fig 3:**
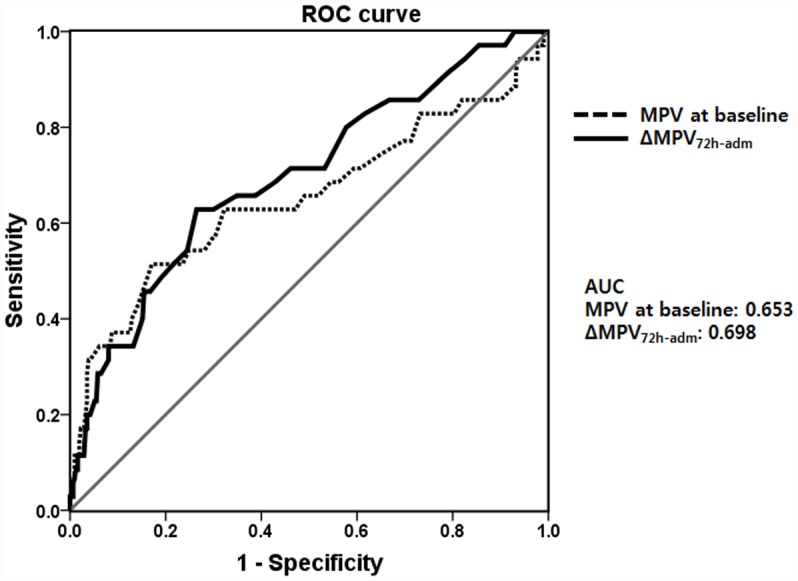
Receiver operating characteristic (ROC) curves of baseline MPV and ΔMPV_72h-adm_ for 28-day all-cause mortality. Area under the curve (AUC) of baseline MPV and ΔMPV_72h-adm_ were 0.653 and 0.698, respectively.

Among the 345 patients with severe sepsis and/or septic shock, 35 (10.1%) died within 28 days after ED admission. Univariate Cox regression analysis revealed that ΔMPV_72h-adm_, male sex, APACHE II and SOFA score, RRT, platelet count, CRP level, lactate level, and infection site were significantly associated with an increased risk of 28-day all-cause mortality (Table 4). On the contrary, higher serum albumin level correlated with a lower risk of 28-day all-cause mortality (Table 4). In multivariate analysis, ΔMPV_72h-adm_ still remained a significant independent risk factor of 28-day all-cause mortality, even after adjusting for age; sex; BMI; SOFA score; RRT; platelet count; and CRP, albumin, and lactate levels (HR, 1.45; 95% CI, 1.02–2.05; *P* = 0.040 in Model 1) (Table 5). Further adjustment of Model 1 for infection site (Model 2) did not attenuate the significant prognostic value of ΔMPV_72h-adm_ on 28-day mortality risk (HR, 1.44; 95% CI, 1.01–2.06; *P* = 0.044) (Table 5). In addition, SOFA score (HR, 1.31; 95% CI, 1.08–1.58; *P* = 0.006) and albumin (HR, 0.45; 95% CI, 0.26–0.80; *P* = 0.006) were still significantly associated with 28-day all-cause mortality in the final multivariate model (Table 6). Moreover, we repeated the analysis with ΔMPV_36h-adm_ (MPV at 36 hours—MPV at baseline). Twelve patients were alive at 36 hours after hospital admission and data of MPV at 36 hours were available in 8 patients. Therefore, we performed multivariate cox regression analysis in order to investigate the association between 28-day all-cause mortality and ΔMPV_36h-adm_, including with these patients. Univariate analysis showed that ΔMPV_36h-adm_ was significantly associated with an increased risk of 28-day all-cause mortality (HR, 1.60; 95% CI, 1.13–2.26; *P* = 0.008). However, its significance was attenuated after adjusting for other covariates in multivariate analysis (data not shown). Receiver operating characteristic (ROC) curves of baseline MPV and ΔMPV_72h-adm_ for 28-day all-cause mortality are shown in Fig. 3. Area under the curve (AUC) of baseline MPV and ΔMPV_72h-adm_ were 0.653 and 0.698, respectively.

**Table 4:** Univariate Cox proportional hazards analysis for 28-day all-cause mortality.

Variable	HR (95% CI)	*P-*value
**ΔMPV**_**72h-adm**_ **(per 1 fL)**	1.90 (1.36–2.66)	<0.001
**Age (per 1 year)**	1.03 (1.00–1.05)	0.059
**Male (versus female)**	2.77 (1.33–5.76)	0.007
**Charlson Comorbidity Index**	1.02 (0.82–1.28)	0.843
**Body mass index (per 1 kg/m** ^**2**^ **)**	0.92 (0.84–1.01)	0.082
**APACHE II score**	1.14 (1.10–1.18)	<0.001
**SOFA score**	1.48 (1.32–1.65)	<0.001
**RRT (versus no RRT)**	6.48 (3.34–12.59)	<0.001
**Hemoglobin (per 1 g/dL)**	0.95 (0.81–1.10)	0.482
**Platelet (per 1 × 10** ^**3**^ **/mm** ^**3**^ **)**	1.00 (0.99–1.00)	0.010
**CRP (per 1 mg/dL)**	1.00 (1.00–1.01)	0.005
**Albumin (per 1 g/dL)**	0.28 (0.18–0.43)	<0.001
**Lactate (per 1 mmol/L)**	1.19 (1.09–1.29)	<0.001
**Infection site**		
Urinary tract	1.00 (reference)	—
Intra-abdominal or other	3.59 (0.80–16.06)	0.094
Lung (pneumonia)	9.13 (2.10–39.73)	0.003
Multiple sites	9.11 (1.77–46.98)	0.008

HR, hazard ratio; CI, confidence interval; ΔMPV_72h-adm_, mean platelet volume at 72 hours—mean platelet volume at baseline; APACHE II, Acute Physiology and Chronic Health Evaluation II; SOFA, Sequential Organ Failure Assessment; RRT, renal replacement therapy; CRP, C-reactive protein.

**Table 5:** Multivariate Cox proportional hazards analysis for 28-day all-cause mortality.

Cox model	ΔMPV_72h-adm_ (per 1 fL)	
	HR (95% CI)	*P* value
**Unadjusted**	1.90 (1.36–2.66)	<0.001
**Model 1**	1.45 (1.02–2.05)	0.040
**Model 2**	1.44 (1.01–2.06)	0.044

Unadjusted: crude relative risk.

Model 1: adjusted for age, sex, body mass index, Sequential Organ Failure Assessment score, renal replacement therapy, platelet count, C-reactive protein level, albumin level, and lactate level.

Model 2: model 1 plus adjustment for infection site.

ΔMPV_72h-adm_, mean platelet volume at 72 hours—mean platelet volume at baseline; HR, hazard ratio; CI, confidence interval.

**Table 6:** Multivariate Cox proportional hazards analysis for 28-day all-cause mortality (Model 2).

Variable	HR (95% CI)	*P-*value
**ΔMPV**_**72h-adm**_ **(per 1 fL)**	1.44 (1.01–2.06)	0.044
**Age (per 1 year)**	1.00 (0.97–1.03)	0.948
**Male (versus female)**	1.12 (0.44–2.88)	0.811
**Body mass index (per 1 kg/m** ^**2**^ **)**	0.94 (0.84–1.06)	0.310
**SOFA score**	1.31 (1.08–1.58)	0.006
**RRT (versus no RRT)**	1.80 (0.81–3.99)	0.147
**Platelet (per 1 × 10** ^**3**^ **/mm** ^**3**^ **)**	1.00 (1.00–1.00)	0.690
**CRP (per 1 mg/dL)**	1.00 (1.00–1.00)	0.516
**Albumin (per 1 g/dL)**	0.45 (0.26–0.80)	0.006
**Lactate (per 1 mmol/L)**	0.99 (0.87–1.13)	0.870
**Infection site**		
Urinary tract	1.00 (reference)	—
Intra-abdominal or other	1.87 (0.38–9.16)	0.440
Lung (pneumonia)	3.77 (0.75–18.95)	0.107
Multiple sites	3.86 (0.68–21.85)	0.127

HR, hazard ratio; CI, confidence interval; ΔMPV_72h-adm_, mean platelet volume at 72 hours—mean platelet volume at baseline; SOFA, Sequential Organ Failure Assessment; RRT, renal replacement therapy; CRP, C-reactive protein.

## Discussion

Severe sepsis and/or septic shock are major healthcare problems affecting millions of people worldwide each year [30,31]. The mortality rates of these conditions are 25% to 80%, depending on illness severity, and the number of occurrences and the severity of organ failure [31,32]. Therefore, a number of initiatives to reduce worldwide sepsis-associated mortality, such as the Surviving Sepsis Campaign that includes the tenets of early hemodynamic optimization (EGDT), have been implemented to overcome this devastating disease [33–35]. Moreover, early detection of progressive severe sepsis and/or septic shock would be not only useful for risk stratification in allocating resources, but also helpful in monitoring treatment efficacy and disease progress.

The present study is a prospective clinical investigation into the prognostic value of changes in MPV in patients receiving a standardized resuscitation algorithm (i.e., EGDT) for severe sepsis and/or septic shock. The main findings of this study are as follows. First, we verified the increase in MPV during the first 72 hours after hospital admission and found a steeper MPV increase in non-survivors than in survivors. This result suggests that although the changes in MPV occurred in a similar direction in both groups, the increased width was greater in non-survivors than in survivors. Second, we revealed that ΔMPV_72h-adm_ is an independent risk factor of 28-day all-cause mortality, in patients with severe sepsis and/or septic shock, even after adjusting for plausible confounding variables. To our knowledge, this study is the first to report a significant association between change in MPV and all-cause mortality in a homogenous group of patients with sepsis.

During the clinical course of sepsis, platelets exhibit diverse characteristics on a wide continuum from hyperactivation to exhaustion, and thrombocytopenia occurs frequently, implying that platelets are involved in pathophysiology beyond hemostatic function [17,18,21]. Sepsis-associated thrombocytopenia has been explained by impaired central platelet production and peripheral overconsumption and/or destruction, but the precise mechanism remains undetermined [18,21]. Generally, platelet counts in patients with sepsis markedly decrease during the first 4 days of hospital admission [17], and the inverse relationship between platelet count and MPV has been described frequently in physiologic and some pathologic conditions [3,4]. We also observed this inverse relationship in the present study. Although these changes of platelet indices in critically ill patients have been investigated in previous studies, the mechanism underlying the relationship between these derangements and mortality has not yet been clarified. Thus, we investigated MPV among the various platelet indices in patients with sepsis, focusing particularly on change in MPV. Several reports have demonstrated that MPV increases in septic milieus. Dastugue *et al*. [36] reported an increase in MPV in patients with shock-related thrombocytopenia. Van der Lelie and Von dem Borne [22] showed a higher MPV in patients with sepsis than in patients with localized infection and suggested that an increase of MPV in patients with bacterial infection could indicate the occurrence of septicemia. Becchi *et al*. [23] examined the trends of MPV and platelet count during the course of sepsis in a small population and found that the average MPV gradually increased in non-survivors, whereas it decreased in survivors. Furthermore, in studies of septic animal models, MPV increased after the induction of sepsis, whereas platelet count changed inversely [37,38]. These results are consistent with the present study findings, and together, these data suggest that continuous monitoring of changes in MPV may play a role in risk stratification of patients with severe sepsis and/or septic shock.

Although there are several contradictory observations [11,14], the nature of MPV as an inflammatory marker has been suggested by previous studies, which have demonstrated a correlation between a higher MPV and active inflammatory disease as aforementioned [12,13,15]. Furthermore, recent advances in the quantification of laboratory markers may demonstrate more firmly that MPV is a reflection of both proinflammatory and prothrombotic conditions, where thrombopoietin and numerous inflammatory cytokines, such as interleukin (IL) -1, -3, and-6 and tumor necrosis factor-α (TNF-α), regulate thrombopoiesis [3]. Larger platelets, indicating an increased MPV, are functionally, metabolically, and enzymatically more active than smaller ones. Because larger platelets have more intracellular thromboxane A_2_ and increased levels of procoagulant surface proteins, such as P-selectin and glycoprotein IIIa, they present a greater prothrombotic potential [5]. Moreover, inflammation by itself can induce procoagulant changes and facilitate embolization, which is one of the major causes of death in patients with systemic bacterial infection [39]. Taken together, MPV can be speculated as an integrative measure of the detrimental processes of inflammation and hypercoagulable state in critical illness and thus, the association between increased MPV and mortality in patients with sepsis can be partially explained by this notion.

Several studies reported the relationship between MPV and renal dysfunction, recently [40,41]. In these studies, MPV was significantly increased with progression of chronic kidney disease and independently associated with GFR. These are in line with our result. Actually, Pearson correlation analysis showed a significant association between baseline MPV and creatinine (r = 0.141, *P* = 0.009). Therefore, an interaction between uremia and MPV may exist. However, we could find that ΔMPV_72h-adm_ was a significantly useful marker for predicting 28-day all-cause mortality even after adjustment for requirement of RRT (Table 5). Moreover, ΔMPV_72h-adm_ was still a significant predictable marker after adjusting AKI episodes (versus non-episode of AKI, HR 1.45; 95% CI 1.010–2.07; *P* = 0.044). In the future, we need further study for the interaction between uremia and MPV value.

This study has several limitations. First, patients were enrolled from a single medical center in South Korea; thus, it is somewhat difficult to generalize the results. Second, we could not thoroughly investigate the previous use of anti-platelet agents and smoking status, which are known to affect MPV [3]. The record of previous use (within 1 week of enrollment) of anti-platelet agent or NSAIDs could be checked only in 134 of 345 patients (38.8%). Among these patients, there was no significant difference between survivors and non-survivors in the use of medications (16.5% vs. 19.6%, *P* = 0.785). However, we considered that there is a limitation to interpret these results inductively. Moreover, even after adjusting the modifying factors in the statistical analyses, the risk relationship between MPV and poor clinical outcome may still be subject to residual confounding. Finally, we could not explain the mechanisms triggering changes in MPV, which were different in amount between survivors and non-survivors, exactly. The investigation and application of additional markers, such as IL-1, IL-3, IL-6, TNF-α, and thrombopoietin, may further elucidate this issue regarding a cause-and-effect relationship. Therefore, further research is required to determine the precise mechanisms underlying the association between MPV and mortality in critically ill patients.

Despite these limitations, the main strength of this study is that a relatively large number of patients were included from a single center; therefore, all study patients underwent similar decision-making for critical care with EGDT. Moreover, very few data sets were missing. We also performed a sensitivity analysis because platelet transfusion might influence platelet indices, including MPV, and thus modify the relationship between the changes in MPV and all-cause mortality. When restricting the analysis to patients who did not receive platelet transfusion within 72 hours after ED admission, we also found similar results (HR 2.19; 95% CI 1.28–3.74; *P* = 0.004 in multivariate analysis with adjusting same variables of Model 2).

## Conclusions

We revealed a greater increase of MPV in non-survivors of severe sepsis and/or septic shock compared with survivors during the first 72 hours after hospitalization and found that an increase in MPV from baseline is an independent risk factor for 28-day all-cause mortality. Although further studies are needed to elucidate the role of changes in MPV as a risk factor in patients with severe sepsis and/or septic shock, MPV can be used as an additional and complementary maker with several established measures of illness severity such as SOFA score, APACHE II score, CRP, albumin, and lactate. Moreover, repeating measurement of MPV may be helpful to predict the prognosis of patients with severe sepsis and/or septic shock. Guardedly, we suggest that physicians should be more attentive to septic patients with higher baseline MPV and tendency of increase in MPV.
